# Thromboembolic Risk in Patients With Pneumonia and New-Onset Atrial Fibrillation Not Receiving Anticoagulation Therapy

**DOI:** 10.1001/jamanetworkopen.2022.13945

**Published:** 2022-05-26

**Authors:** Mette Søgaard, Flemming Skjøth, Peter B. Nielsen, Jesper Smit, Michael Dalager-Pedersen, Torben B. Larsen, Gregory Y. H. Lip

**Affiliations:** 1 Department of Cardiology, Aalborg University Hospital, Aalborg, Denmark; 2 Aalborg Thrombosis Research Unit, Department of Clinical Medicine, Faculty of Health, Aalborg University, Aalborg, Denmark; 3 Unit for Clinical Biostatistics, Aalborg University Hospital, Aalborg, Denmark; 4 Department of Infectious Diseases, Aalborg University Hospital, Aalborg, Denmark; 5 Liverpool Centre for Cardiovascular Science, University of Liverpool and Liverpool Heart & Chest Hospital, Liverpool, United Kingdom

## Abstract

**Importance:**

New-onset atrial fibrillation (AF) is commonly reported in patients with severe infections. However, the absolute risk of thromboembolic events without anticoagulation remains unknown.

**Objective:**

To investigate the thromboembolic risks associated with AF in patients with pneumonia, assess the risk of recurrent AF, and examine the association of initiation of anticoagulation therapy with new-onset AF.

**Design, Setting, and Participants:**

This population-based cohort study used linked Danish nationwide registries. Participants included patients hospitalized with incident community-acquired pneumonia in Denmark from 1998 to 2018. Statistical analysis was performed from August 15, 2021, to March 12, 2022.

**Exposures:**

New-onset AF.

**Main Outcomes and Measures:**

Thromboembolic events, recurrent AF, and all-cause death. Estimated risks were calculated for thromboembolism without anticoagulation therapy, new hospital or outpatient clinic contact with AF, initiation of anticoagulation therapy, and all-cause death at 1 and 3 years of follow-up. Death was treated as a competing risk, and inverse probability of censoring weights was used to account for patient censoring if they initiated anticoagulation therapy conditioned on AF.

**Results:**

Among 274 196 patients hospitalized for community-acquired pneumonia, 6553 patients (mean age [SD], 79.1 [11.0] years; 3405 women [52.0%]) developed new-onset AF. The 1-year risk of thromboembolism was 0.8% (95% CI, 0.8%-0.8%) in patients without AF vs 2.1% (95% CI, 1.8%-2.5%) in patients with new-onset AF without anticoagulation; this risk was 1.4% (95% CI, 1.0%-2.0%) among patients with AF with intermediate stroke risk and 2.8% (95% CI, 2.3%-3.4%) in patients with AF with high stroke risk. Three-year risks were 3.5% (95% CI, 2.8%-4.3%) among patients with intermediate stroke risk and 5.3% (95% CI, 4.4%-6.5%) among patients with high stroke risk. Among patients with new-onset AF, 32.9% (95% CI, 31.8%-34.1%) had a new hospital contact with AF, and 14.0% (95% CI, 13.2%-14.9%) initiated anticoagulation therapy during the 3 years after incident AF diagnosis. At 3 years, the all-cause mortality rate was 25.7% (95% CI, 25.6%-25.9%) in patients with pneumonia without AF vs 49.8% (95% CI, 48.6%-51.1%) in patients with new-onset AF.

**Conclusions and Relevance:**

This cohort study found that new-onset AF after community-acquired pneumonia was associated with an increased risk of thromboembolism, which may warrant anticoagulation therapy. Approximately one-third of patients had a new hospital or outpatient clinic contact for AF during the 3-year follow-up, suggesting that AF triggered by acute infections is not a transient, self-terminating condition that reverses with resolution of the infection.

## Key Points

**Question:**

What is the risk of thromboembolism and recurrent atrial fibrillation after infection-related atrial fibrillation?

**Findings:**

In this cohort study including 274 196 patients with pneumonia, of whom 6553 developed atrial fibrillation, the 1-year risk of thromboembolism was 0.8% in patients without atrial fibrillation vs 2.1% in patients with atrial fibrillation. Among patients with new-onset atrial fibrillation, 32.9% had a recurrent hospital contact with atrial fibrillation and 14.0% initiated anticoagulation therapy during 3-year follow-up.

**Meaning:**

These findings suggest that the concept of infection-related atrial fibrillation as a transient condition needs reconsideration given the high risks of recurrence combined with risks of thromboembolism that may warrant anticoagulation therapy.

## Teaser

This cohort study investigates the thromboembolic risks associated with atrial fibrillation (AF) in patients with pneumonia, assesses the risk of recurrent AF, and examines the association of initiation of anticoagulation therapy with new-onset AF.

## Introduction

Pneumonia and atrial fibrillation (AF) are leading causes of morbidity and mortality worldwide.^1,2,3^ Pneumonia is the most common medical diagnosis responsible for hospitalizations in the US.^4^ AF is the most common cardiac arrhythmia and carries up to a 5-fold increased risk of stroke.^5,6^ Pneumonia and AF often coexist, and new-onset AF is a common complication occurring in 4.7% to 9.5% of patients with pneumonia.^7,8,9^

A prevailing thought has been that AF triggered by acute infection is a transient and self-terminating condition that reverses with resolution of infection. However, mounting evidence suggest that AF frequently recurs, carrying an increased risk of stroke.^10,11,12,13^ Stroke risk in patients with infections and AF has been shown to exceed the risk of both the general population with AF^10,11,12^ and patients with infections without AF.^13^ Nonetheless, guidelines do not provide clear recommendations regarding the role of oral anticoagulant (OAC) therapy to mitigate stroke risk in this context. The decision to initiate OAC therapy should be based on the expected net clinical benefit of OAC therapy, which tracks closely with the absolute risk of stroke and bleeding in patients not receiving OAC therapy. Patients at low stroke risk may have little to no or negative net clinical benefit from therapy. Decision analyses have indicated that the threshold at which OAC treatment yields a net clinical benefit is a stroke risk between 1% to 2% per year.^14,15,16^ However, there is a paucity of data on the absolute thromboembolic risk associated with AF triggered by infections to guide the treatment decision among patients with pneumonia and incident AF.

This nationwide cohort study in Denmark sought to investigate the risks of arterial thromboembolism in patients with new-onset AF after community-acquired pneumonia without anticoagulation therapy. In secondary analyses, we sought to clarify the concept of postinfection new-onset AF as a transient condition by estimating risks of recurrent hospital or outpatient clinic contact with AF, OAC therapy initiation, and all-cause mortality.

## Methods

This cohort study included all patients hospitalized with community-acquired pneumonia in Denmark from January 1998 through June 2018. The study was approved by the Danish Data Protection Agency. Registry studies do not require ethical approval or informed consent in Denmark. The data were provided by the Danish Health Data Authority. We followed the Strengthening the Reporting of Observational Studies in Epidemiology (STROBE) reporting guideline for cohort studies.^17^

### Setting and Data Sources

The source population included the entire population of Denmark (5.6 million inhabitants). Denmark has a tax-funded universal health care system, with equal access to hospitals and primary care for all residents and partial reimbursement of medication costs. A unique civil registration number, assigned to all residents, is used to track individual health services in nationwide registries. This study was based on linkage between 3 registries: (1) the Danish National Patient Register, which includes information on all discharge diagnoses from Danish hospitals^18^; (2) The Civil Registration system containing information about age, sex, and vital status^19^; and (3) The Danish National Prescription Registry storing information about all claimed prescriptions from Danish people.^20^

### Study Population

We focused on pneumonia because it is a well-characterized prevalent infection that has been consistently linked with cardiovascular disease, including AF.^21,22^ We used the National Patient Register to identify all patients aged at least 18 years at incident primary inpatient pneumonia diagnosis. To exclude confounding from underlying conditions and procedures, we restricted the study to patients with community-acquired pneumonia. Accordingly, we excluded all patients with hospital contact (inpatient or ambulatory) within the 30 days before pneumonia admission. To ensure lookback time for prevalent AF diagnoses, we further excluded patients not residing in Denmark for at least 2 years before pneumonia diagnosis at hospital discharge (index date).

Because we focused on new-onset AF following pneumonia, we screened the pneumonia cohort for status of AF according to diagnoses and prescriptions. We excluded patients with prior AF diagnoses and patients with prior experience of any OAC treatment within 180 days before index date. To investigate risk of outcomes in patients not receiving OAC, we further excluded patients who developed a study end point, initiated OAC treatment, or died during the landmark period of 30 days after discharge from pneumonia hospitalization (eFigure in the Supplement).

### Outcomes and Comorbidity

We studied the at-risk population of patients who were alive and not receiving anticoagulation therapy at the landmark time point of 30 days after the index date and categorized patients according to whether they received a diagnosis of new-onset AF during the landmark period. Follow-up started at the end of the 30-day landmark for a maximum of 3 years, with administrative censoring at December 31, 2018, or at emigration. The primary outcome was a record of arterial thromboembolic event defined as ischemic stroke and/or systemic arterial embolism. Given the severity of the diagnosis of thromboembolism, we only considered events where thromboembolism was the reason for hospital admission; hence, we did not consider secondary and outpatient diagnoses. Validation studies of diagnoses in the National Patient Registry have shown high positive predictive values for AF (approximately 95%), ischemic stroke (approximately 97%),^18^ and other cardiovascular diagnoses^23^ and comorbidities.^18^ Secondary outcomes included recurrent hospital or outpatient clinic contact with AF, OAC therapy initiation, and all-cause mortality. For code definitions, see eTable 1 in the Supplement.

We combined covariate information into the CHA_2_DS_2_-VASc (congestive heart failure or left ventricular ejection fraction ≤40%; hypertension; age ≥75 years; diabetes; stroke, transient ischemic attack, or thromboembolism history; vascular disease; age 65-74 years; and female sex) score^24^ as a measure of stroke risk and a HAS-BLED (hypertension, abnormal kidney or liver function, stroke, bleeding history or predisposition, labile international normalized ratio, elderly [age ≥65 years], and drugs or alcohol concomitantly) score^25^ as a measure of bleeding risk (see score definitions in eTable 2 in the Supplement). Baseline stroke risk according to CHA_2_DS_2_-VASc score was categorized on the basis of assigned points: low risk (0, no risk factors when disregarding female sex as a lone risk factor), intermediate risk (1-2 risk factors), and high risk (≥3 risk factors) where points for female sex were not considered.

### Statistical Analysis

Baseline characteristics at pneumonia diagnosis were summarized using means and SDs for continuous measures and percentages for categorical measures. We used time-to-event analyses to analyze outcome risks measuring time at risk from the 30-day landmark to outcome of interest, emigration, death, or end of follow-up, whichever came first. We investigated the absolute risk of events, taking into account the competing risk of death, and hereby incorporating the diminishing at-risk population under the expectation of a relatively high risk of death and number of outcomes.^26^ Specifically, we used the Aalen-Johansen estimator to estimate risk development for all end points over time, assuming death as competing risk.^24^ Cause-specific Cox regression with adjustment for inclusion period, sex, and age (included as restricted cubic spline) was used to assess the risk of arterial thromboembolism comparing patients with new-onset AF vs those without new-onset AF.

For analyses of arterial thromboembolism in patients with new-onset AF, we censored patients when they started OAC therapy to estimate risks had these patients not initiated anticoagulation and to avoid potential structural selection bias between groups (ie, those at higher risk would be more likely to initiate OAC treatment). We used an inverse probability of censoring weighted (IPCW) analysis to handle informative censoring related to OAC therapy initiation.^27^ Risk of OAC therapy initiation during follow-up was estimated using a Fine-Grey regression model with death as competing risk and accounting for baseline and time-varying confounding factors, including age, sex, heart failure, hypertension, stroke, diabetes, vascular disease, use of diuretics, renin-angiotensin system inhibitors, β-blocker, or calcium-channel blocker. Recurrent hospital or outpatient clinic contact with AF during follow-up was also included as a time-varying covariate as it may trigger OAC therapy initiation. To allow for time-varying covariates, the data set was structured in the counting process style with multiple rows of data per patient by splitting the follow-up into 7-day periods and evaluate covariates and outcomes at every interval. The IPCW given by the inverse of the survival function of the Fine-Grey model for the outcome of OAC initiation was subsequently estimated by a weighted Cox proportional hazards model.^28^ To account for repeated observations, we applied a sandwich estimator of the covariance matrix. To reduce influence of extreme IPCWs, the weights were stabilized by the conditional probability of treatment initiation only, given that the inclusion period accounted for the competing risk of death. The 95% CIs for arterial thromboembolic risk were estimated by bootstrapping with 1000 samples. Because of small sample size and few events, we decided post hoc not to use IPCW methods for patients with low stroke risk, because this may reduce the fit of the censoring model and precision of estimated weights. IPCW methods were not used for assessment of secondary outcomes, because patients were not censored according to OAC therapy use.

In sensitivity analyses, we examined potential outcomes of changing guidelines for OAC treatment. First, we stratified the baseline hazard function by inclusion period (1998-2003, 2004-2008, 2009-2013, and 2014-2018) and included interactions between inclusion period and all covariates when estimating IPCW weights. Next, we examined outcome risks with restriction to the most recent period, 2014 to 2018. Finally, although aspirin does not provide optimal stroke protection in patients with AF, there is evidence of some benefit in terms of lower stroke rates compared with no treatment.^29^ Therefore, we conducted a sensitivity analysis excluding patients with baseline aspirin treatment to ascertain whether this would affect thromboembolic risk.

Statistical tests represent 2-sided hypotheses and a 5% level (*P* < .05) was used to evaluate significance. Analyses were performed using Stata/MP statistical software version 16 (StataCorp) and R statistical software version 3.1.1 (The R Project for Statistical Computing). Statistical analysis was performed from August 15, 2021, to March 12, 2022.

## Results

### Baseline Characteristics

From January 1, 1998, to September 30, 2018, we identified 293 051 adult patients with incident hospitalized community-acquired pneumonia, of whom 11 107 (3.8%) developed new-onset AF (eFigure in the Supplement). After exclusion of patients who died, initiated OAC therapy, or experienced a thromboembolic event, the 30-day landmark population included 274 196 patients, of whom 6553 (mean age [SD], 79.1 [11.0] years; 3405 women [52.0%]) had new-onset AF (eFigure in the Supplement and Table 1). The new-onset AF group was characterized by higher stroke risk and greater prevalence of cardiovascular and noncardiovascular comorbidities compared with patients with pneumonia without AF. Most patients (6162 of 6443 patients [94%]) with new-onset AF had intermediate or high CHA_2_DS_2_-VASc score.

**Table 1.** Baseline Characteristics of Patients Not Receiving Anticoagulation Therapy With Incident Community-Acquired Pneumonia According to Development of New-Onset AF

Characteristic	Patients, No. (%)	
	Pneumonia without AF (n = 267 643)	New-onset AF (n = 6553)
Period of study inclusion		
1998-2003	66 167 (24.7)	1984 (30.3)
2004-2008	56 478 (21.1)	1725 (26.3)
2009-2013	62 182 (23.2)	1840 (28.1)
2014-2018	82 816 (30.9)	1004 (15.3)
Hospital stay, median (IQR), d	4.9 (2.3-8.9)	9.0 (5.1-17.0)
Sex		
Female	138 011 (51.6)	3405 (52.0)
Male	129 632 (48.4)	3148 (48.0)
Age, mean (SD), y	63.8 (18.6)	79.1 (11.0)
Comorbidity		
CHA_2_DS_2_-VASc score, mean (SD)	2.1 (1.6)	3.2 (1.4)
Stroke risk categories^a^		
Low	96 492 (36.1)	391 (6.0)
Intermediate	97 248 (36.3)	2584 (39.4)
High	73 903 (27.6)	3578 (54.6)
HAS-BLED score, mean (SD)	1.4 (1.2)	2.2 (1.1)
Prior bleeding	26 005 (9.7)	879 (13.4)
Kidney disease	10 033 (3.7)	396 (6.0)
Alcohol-related disease	2419 (0.9)	75 (1.1)
Heart failure	18 984 (7.1)	1364 (20.8)
Diabetes	30 219 (11.3)	837 (12.8)
Vascular disease	24 446 (9.1)	890 (13.6)
Hypertension	68 000 (25.4)	2601 (39.7)
Prior ischemic stroke	21 248 (7.9)	803 (12.3)
Chronic obstructive pulmonary disease	45 767 (17.1)	1417 (21.6)
Cancer	39 556 (14.8)	1174 (17.9)
Ischemic heart disease	37 508 (14.0)	1451 (22.1)
Myocardial infarction	19 170 (7.2)	734 (11.2)
Venous thromboembolism	9297 (3.5)	248 (3.8)
Comedications		
Digoxin	4323 (1.6)	840 (12.8)
Clopidogrel	10 149 (3.8)	253 (3.9)
Aspirin	57 439 (21.5)	2345 (35.8)
Renin-angiotensin system inhibitors (angiotensin-converting enzyme inhibitors or angiotensin-receptor blockers)	57 902 (21.6)	1764 (26.9)
Statins	47 210 (17.6)	1061 (16.2)
Nonsteroidal anti-inflammatory drug	64 862 (24.2)	1456 (22.2)
β-blocker	35 839 (13.4)	1363 (20.8)
Calcium	40 889 (15.3)	1488 (22.7)
Loop diuretics	42 414 (15.8)	1925 (29.4)
Nonloop diuretics	59 292 (22.2)	2204 (33.6)
Proton-pump inhibitors	57 061 (21.3)	1414 (21.6)
Antibiotics within 30 d	91 831 (34.3)	1760 (26.9)

Abbreviations: AF, atrial fibrillation; CHA_2_DS_2_-VASc, congestive heart failure or left ventricular ejection fraction ≤40%; hypertension; age ≥75 years; diabetes; stroke, transient ischemic attack, or thromboembolism history; vascular disease; age 65-74 years; and female sex; HAS-BLED, hypertension, abnormal kidney or liver function, stroke, bleeding history or predisposition, labile international normalized ratio, elderly (age ≥65 years), and drugs or alcohol concomitantly.

^a^ Baseline stroke risk according to CHA_2_DS_2_-VASc score was categorized according to assigned points: low risk (0, no risk factors when disregarding female sex as a lone risk factor), intermediate risk (1-2 risk factors), and high risk (≥3 risk factors) where points for female sex were taken into account.

### Arterial Thromboembolism

During the 3 years of follow-up, rates of thromboembolic events were significantly higher in patients with new-onset AF than in patients without AF (2.21 cases per 100 person-years [95% CI, 1.97-2.49 cases per 100 person-years] vs 0.83 per 100 person-years [95% CI, 0.81-0.86 cases per 100 person-years]); adjusted hazard ratio [HR], 1.49 [95% CI, 1.32-1.69]) (Table 2 and Figure). When we stratified patients with new-onset AF by baseline stroke risk, we observed fewer than 5 thromboembolic events in patients at low stroke risk throughout follow-up. During 1 year of follow-up, there were 38 thromboembolic events among patients with new-onset AF and intermediate stroke risk (IPCW weighted rate of 1.88 events per 100 person-years [95% CI, 1.36-2.68 events per 100 person-years]) and 99 events among patients at high risk (weighted rate of 3.87 events per 100 person-years [95% CI, 3.17-4.76 events per 100 person-years]). At 3 years, the weighted incidence rate was 1.75 events per 100 person-years (95% CI, 1.41-2.20 events per 100 person-years) for patients with intermediate stroke risk and 3.07 events per 100 person-years (95% CI, 2.55-3.70 events per 100 person-years) for patients with high stroke risk. The attenuating rate estimate for 3-year vs 1-year follow-up reflect the diminishing at-risk population due to right censoring (ie, end of study, OAC therapy initiation, or death).

**Table 2.** Number of Events, Crude, and Weighted Rate of Thromboembolic Events in Patients Not Receiving Anticoagulation Therapy According to Presence of New-Onset AF

Strata	1-y Follow-up			3-y Follow-up		
	Events, No.	Rate, events/100 person-years (95% CI)		Events, No.	Rate, events/100 person-years (95% CI)	
		Crude	Weighted^a^		Crude	Weighted^a^
Pneumonia without AF	2059	0.93 (0.89-0.97)	NA	4982	0.83 (0.81-0.86)	NA
Pneumonia with new-onset AF	137^b^	2.80 (2.37-3.31)	2.82 (2.38-3.37)	272^b^	2.21 (1.97-2.49)	2.29 (1.99-2.65)
Baseline stroke risk^c^						
Low	<5^b^	0.30 (0.04-2.14)	NA^d^	<5^b^	0.31 (0.10-0.97)	NA^d^
Intermediate	38	1.91 (1.39-2.63)	1.88 (1.36-2.68)	89	1.71 (1.39-2.11)	1.75 (1.41-2.20)
High	99	3.80 (3.12-4.63)	3.87 (3.17-4.76)	183	2.92 (2.53-3.38)	3.07 (2.56-3.70)

Abbreviations: AF, atrial fibrillation; NA, not applicable.

^a^ Patients with new-onset AF were censored when they started anticoagulation therapy and were weighted by the inverse of the probability of being censored.

^b^ As required by Danish data protection law, percentages and counts were suppressed for observations with fewer than 5 incidents to prevent disclosure of potentially identifiable information. Accordingly, the number of events among patients at low risk is not included in the overall number of events.

^c^ Among patients with new-onset AF, baseline stroke risk is determined according to CHA_2_DS_2_-VASc score (congestive heart failure or left ventricular ejection fraction ≤40%; hypertension; age ≥75 years; diabetes; stroke, transient ischemic attack, or thromboembolism history; vascular disease; age 65-74 years; and female sex) categorized by assigned points: low risk (0, no risk factors when disregarding female sex as a lone risk factor), intermediate risk (1-2 risk factors), and high risk (≥3 risk factors) where points for female sex were not taken into account.

^d^ Because of low sample size and few events (<5 events), weighted rates were not estimated for patients at low baseline risk of stroke (CHA_2_DS_2_-VASc score of 0 to 1 when disregarding female sex as a lone risk factor).

**Figure.**
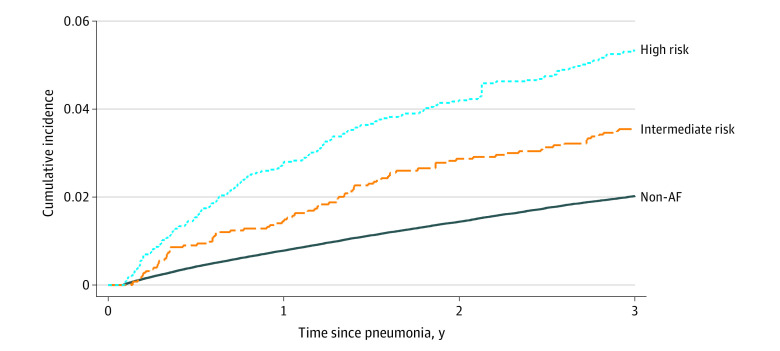
Risk of Thromboembolic Events in Patients Not Receiving Anticoagulation Therapy After Community-Acquired Pneumonia According to Development of New-Onset Atrial Fibrillation (AF) Patients with new-onset AF were censored when they started anticoagulation therapy and death was treated as a competing risk. Patient data were weighted by the inverse probability of censoring.

At 1 year, the risk of thromboembolism was 0.8% (95% CI, 0.8%-0.8%) in patients without AF vs 2.1% (95% CI, 1.8%-2.5%) in patients with new-onset AF not receiving anticoagulation. Among patients with new-onset AF, the risk was 1.4% (95% CI, 1.0%-2.0%) in patients at intermediate stroke risk and 2.8% (95% CI, 2.3%-3.4%) in high-risk patients (Table 3). At 3-year follow-up, corresponding risks were 3.5% (95% CI, 2.8%-4.3%) in patients with intermediate stroke risk and 5.3% (95% CI, 4.4%-6.5%) in patients with high stroke risk (Figure).

**Table 3.** Absolute Risk of Thromboembolism in Patients Not Receiving Anticoagulation Therapy According to Presence of New-Onset AF

Strata	Thromboembolism risk, % (95% CI)^a^	
	1-y Follow-up	3-y Follow-up
Pneumonia without AF	0.8 (0.8-0.8)	2.0 (2.0-2.1)
Pneumonia with new-onset AF	2.1 (1.8-2.5)	4.4 (3.7-5.0)
Baseline stroke risk^b^		
Low	NA^c^	NA^c^
Intermediate	1.4 (1.0-2.0)	3.5 (2.8-4.3)
High	2.8 (2.3-3.4)	5.3 (4.4-6.5)

Abbreviations: AF, atrial fibrillation; NA, not applicable.

^a^ Death was treated as a competing risk. Patients with new-onset AF were censored if they started anticoagulation therapy and were weighted by the inverse of the probability of being censored.

^b^ Among patients with new-onset AF, baseline stroke risk was determined according to CHA_2_DS_2_-VASc score (congestive heart failure or left ventricular ejection fraction ≤40%; hypertension; age ≥75 years; diabetes; stroke, transient ischemic attack, or thromboembolism history; vascular disease; age 65-74 years; and female sex) assigned points: low risk (0-1, no risk factors when disregarding female sex as a lone risk factor), intermediate risk (1-2 risk factors), and high risk (≥3 risk factors) where points for female sex were not taken into account.

^c^ Because of low sample size and few events (<5 events), inverse probability of censoring methods was not applied for patients at low baseline risk of stroke (CHA_2_DS_2_-VASc score of 0-1 when disregarding female sex as a lone risk factor).

### Recurrent Hospital or Outpatient Clinic Contact for AF, OAC Therapy Initiation, and Death

Among patients with new-onset AF, 8.7% (95% CI, 8.1%-9.4%) claimed a prescription for OAC therapy during the first year after hospital admission with community-acquired pneumonia, increasing to 14.0% (95% CI, 13.2%-14.9%) at 3 years (Table 4). Concurrently, 6.7% (95% CI, 4.5%-9.4%) to 9.9% (95% CI, 8.7%-11.1%) claimed a prescription for OAC therapy during the first year depending on baseline stroke risk, increasing to 12.5% (95% CI, 11.4%-13.6%) to 16.1% (95% CI, 14.7%-17.6%) at 3 years (Table 4). Mortality rates were 12.1% (95% CI, 12.0%-12.2%) in patients without AF vs 26.3% (95% CI, 25.2%-27.4%) in patients with new-onset AF at 1 year, and 25.7% (95% CI, 25.6%-25.9%) vs 49.8% (95% CI, 48.6%-51.1%) at 3 years.

**Table 4.** Absolute Risk of AF Rehospitalization, Initiation of Anticoagulation Therapy, and Death in Patients With New-Onset AF After Community-Acquired Pneumonia Not Receiving OAC Therapy

Strata	Risk, % (95% CI)					
	AF rehospitalization		OAC initiation		All-cause death^a^	
	1-y Follow-up	3-y Follow-up	1-y Follow-up	3-y Follow-up	1-y Follow-up	3-y Follow-up
New-onset AF	20.5 (19.6-21.5)	32.9 (31.8-34.1)	8.7 (8.1-9.4)	14.0 (13.2-14.9)	26.3 (25.2-27.4)	49.8 (48.6-51.1)
Baseline stroke risk^b^						
Low	16.2 (12.7-20.0)	27.7 (23.3-32.2)	6.7 (4.5-9.4)	14.5 (11.2-18.2)	10.8 (8.1-14.4)	17.6 (14.2-21.9)
Intermediate	19.9 (18.4-21.5)	31.6 (29.8-33.5)	9.9 (8.7-11.1)	16.1 (14.7-17.6)	22.8 (21.3-24.5)	43.7 (41.8-45.7)
High	21.5 (20.1-22.8)	34.3 (32.8-35.9)	8.1 (7.3-9.1)	12.5 (11.4-13.6)	30.4 (29.0-32.0)	57.7 (56.1-59.4)

Abbreviations: AF, atrial fibrillation; OAC, oral anticoagulation.

^a^ Death was treated as a competing risk.

^b^ Among patients with new-onset AF, baseline stroke risk was determined according to CHA_2_DS_2_-VASc score (congestive heart failure or left ventricular ejection fraction ≤40%; hypertension; age ≥75 years; diabetes; stroke, transient ischemic attack, or thromboembolism history; vascular disease; age 65-74 years; and female sex) assigned points: low risk (0-1, no risk factors when disregarding female sex as a lone risk factor), intermediate risk (1-2 risk factors), and high risk (≥3 risk factors) where points for female sex were not taken into account.

### Sensitivity Analyses

One-year and 3-year risks of thromboembolic events among patients with new-onset AF changed little when accounting for temporal changes by stratifying the baseline hazard function by inclusion period and including interactions between inclusion period and all covariates in the Fine-Grey regression model (eTable 3 in the Supplement). Restriction to the years 2014 to 2018 slightly attenuated the thromboembolic risk; 1-year risk of thromboembolic events without anticoagulation was 1.3% in patients at intermediate risk of stroke and 2.5% in high-risk patients. When excluding patients with baseline aspirin treatment the risk of thromboembolic events was 2.1% at 1 year and 3.9% at 3 years.

## Discussion

Among 293 051 patients with community-acquired pneumonia and no history of AF, 3.8% developed AF after pneumonia hospitalization. Without OAC therapy, new-onset AF was associated with a 2.1% 1-year risk of arterial thromboembolic events (1.4% among patients with intermediate stroke risk and 2.8% among patients at high risk). The 3-year risks were 3.5% and 5.3%, respectively. These estimates exceed the general threshold of 1.7% per year for vitamin K antagonists and 0.9% for the non–vitamin K OACs for obtaining a positive net clinical benefit of stroke prevention^14,15,16^ and are considerably higher than risks observed among patients with pneumonia without AF. Among patients with new-onset AF, 32.9% (95% CI, 31.8%-34.1%) had a new hospital or outpatient clinic contact with AF and 14.0% (95% CI, 13.2%-14.9%) initiated OAC during follow-up.

### Comparison With Other Studies

This study expands the evidence suggesting that there is an increased risk of thromboembolism in patients with infection-related AF, and it adds to the notion that new-onset AF should not be regarded as self-limiting and benign. Previous studies have demonstrated similar relative risk of thromboembolism in patients with infection-related AF compared with patients with AF without infection matched by baseline OAC treatment status^10,11,12^ and in patients with infections without AF.^13^ Among patients with infection-related AF, pneumonia was associated with the highest risk of thromboembolic events,^10,13^ but the association has also has been demonstrated in patients with sepsis.^30^ The benefit of OAC therapy was similar between matched patients with infection-related AF and non–infection-related AF; for infection-related AF, the adjusted HR for thromboembolism was 0.75 (95% CI, 0.68-0.83) compared with those without OAC therapy, and for non–infection-related AF the HR was 0.70 (95% CI, 0.63-0.78).^10^ Most previous studies have only reported the relative risks, which does not inform about baseline risk and may obscure the magnitude of the association. In many situations, the absolute risk gives a better representation of the clinical situation, also from the patient’s point of view.^31^ Our study reporting absolute risks and focusing on a specific infection in patients without OAC, therefore, supports and strengthens the validity of previous findings.

### Potential Explanations and Clinical Implications

Several mechanisms explain why infections can trigger AF, including systemic inflammation, electrophysiological disturbances, metabolic imbalances, hypoxia, and dysvolemia.^32^ New-onset AF after pneumonia may identify a subset of patients who have a substrate for thromboembolism regardless of the arrhythmia. A key finding of our study was that the AF episode was not a transient, self-limited event, because approximately one-third of patients had a new hospital AF diagnosis during follow-up. This corroborates previous findings^13,33^ and suggests that restoration of sinus rhythm with resolution of infection may not protect against AF recurrence. In this respect, infection may merely be a stress test that demonstrates the likelihood for future AF. The substantial observed risk of recurrent AF suggests that individuals with new-onset AF in the context of pneumonia may have underlying predisposition to AF. Indeed, patients with AF in our cohort were older and had a high prevalence of comorbidities, both associated with high risk of AF and thromboembolism.

From a clinical point of view, our study has 2 major implications. First, our findings suggest that new-onset AF during pneumonia is a marker of future AF. This implies that vigilance for recurrent AF may be warranted and that attention should be given to each episode of infection-related AF, regardless of its duration, to tailor surveillance in patients developing this complication. Second, our findings suggest that new-onset AF occurring with pneumonia is associated with a risk of thromboembolism that reaches a level where OAC therapy is considered beneficial. The question remains whether these patients should initiate lifelong OAC therapy. Previously, a UK study found that patients with resolved AF remained at higher risk of stroke than patients without AF.^34^ According to current guidelines, patients with non–infection-related AF receive OAC therapy according to their perceived stroke risk rather than the heart rhythm displayed at a particular time.^35,36^ The updated European Society of Cardiology guidelines^35^ state that the clinical pattern of AF (ie, first detected, paroxysmal, persistent, longstanding persistent, or permanent) should not condition the indication to thromboprophylaxis. In the era of non–vitamin K OAC therapy, perhaps it is time for infection-related AF to be treated in the same way as non–infection-related AF, with long-term anticoagulation maintained in patients with manifested clinical stroke risk factors. However, the decision to initiate anticoagulation treatment in the setting of severe infections is complex. High bleeding risk due to systemic activation of the inflammatory response and depletion of coagulation factors and platelets may outweigh the benefits of anticoagulation. Most trials investigating different OAC therapies for stroke prophylaxis excluded patients with AF due to reversible conditions,^37,38,39,40^ and high-quality evidence regarding the role of OAC in patients with infection-related AF is lacking.

### Strengths and Limitations

Our study was strengthened by the completeness of data in a nationwide cohort of patients experiencing incident AF after community-acquired pneumonia. The Danish health care system provides equal access to health care services for all residents regardless of socioeconomic and insurance status. In Denmark, OAC therapy can be purchased only through prescription, and all Danish pharmacies register redeemed prescriptions ensuring complete and accurate registration.

Our study also has limitations. New-onset AF was defined by a record of an *International Statistical Classification of Diseases and Related Health Problems, Tenth Revision* diagnosis, and we may have missed episodes given the known risk of brief, self-terminated AF episodes that may be undetected or unreported. Therefore, our findings may not generalize to very brief, self-terminating AF episodes. We lacked information on duration and number of AF episodes during admission, and detailed classification of strokes as cardioembolic vs other was not feasible.

## Conclusions

Among patients with community-acquired pneumonia, new-onset AF was associated with increased risks of thromboembolism, which may cross the threshold where OAC therapy is considered beneficial. One-third of patients had a new hospital AF diagnosis during follow-up, indicating that the concept of infection-related AF as a transient condition may need reconsideration. These findings may have rhythm monitoring and treatment implications, and improved communication and monitoring of long-term AF risks is warranted.

## Notes

Supplement. **eTable 1.** Definitions on Comorbidity and Concomitant Medication According to ICD-10 Codes and ATC-codes **eTable 2.** Risk Score Definition **eTable 3.** Results of Sensitivity Analyses Among Patients With an Indication for OAC Therapy (i.e., Intermediate or High Baseline Stroke Risk) **eFigure.** Flow Diagram of the Cohort Creation Process
