# Supplementary material for: Thromboembolic Risk in Patients With Pneumonia and New-Onset Atrial Fibrillation Not Receiving Anticoagulation Therapy
Source: JAMA Netw Open. 2022 May 26;5(5):e2213945. doi: 10.1001/jamanetworkopen.2022.13945 (PMC9136621; doi:10.1001/jamanetworkopen.2022.13945)
Supplement: Supplement. — eTable 1. Definitions on Comorbidity and Concomitant Medication According to ICD-10 Codes and ATC-codes eTable 2. Risk Score Definition eTable 3. Results of Sensitivity Analyses Among Patients With an Indication for OAC Therapy (i.e., Intermediate or High Baseline Stroke Risk) eFigure. Flow Diagram of the Cohort Creation Process [file jamanetwopen-e2213945-s001.pdf]

Supplemental Online Content

Søgaard M, Skjøth F, Nielsen PB, et al. Thromboembolic risk in patients with pneumonia and new-onset atrial fibrillation not receiving anticoagulation therapy. *JAMA Netw Open.* 2022;5(5):e2213945. doi:10.1001/jamanetworkopen.2022.13945

eTable 1. Definitions on Comorbidity and Concomitant Medication According to ICD-10 Codes and ATC-codes

eTable 2. Risk Score Definition

eTable 3. Results of Sensitivity Analyses Among Patients With an Indication for OAC Therapy (i.e., Intermediate or High Baseline Stroke Risk)

eFigure. Flow Diagram of the Cohort Creation Process

This supplemental material has been provided by the authors to give readers additional information about their work.

© 2022 Søgaard M et al. *JAMA Network Open.*

eTable 1. Definitions on comorbidity and concomitant medication according to International Classification of Diseases (ICD) codes and Anatomic Therapeutic Chemical (ATC) codes.

Disease	ICD-8 codes	ICD-10 codes	ATC-code
**Study population**			
Pneumonia		J12 J13 J14 J15 J16 J17 J18	
**Exposure**			
Atrial Fibriallation	42793 42794	I48	
**Exclusion criteria**			
Oral anticoagulant use			
Warfarin			B01AA03
Dabigatran			B01AE07
Rivaroxaban			B01AF01
Apixaban			B01AF02
Edoxaban			B01AF03
**Outcomes**			
Ischemic stroke	43300 43309 43409 43499 43601 43690	I63	
Arterial embolism and thrombosis	444	I74	
**Comorbidity**			
Prior bleeding		I850 I864A K226 K228F K250 K252 K254 K256 K260 K262 K264 K266 K270 K272 K274 K276 K280 K282 K284 K286 K290 K298A K625 K638B K638C K661 K838F K868G K920 K921 K922 I60 I61 I62 I690 I691 I692 S063C S064 S065 S066 S068B S068D S141C S141D S141E S241D S241E S241F S341D S341E S341F E078B E274B G951A I312 I319A I230 J942 M250 R04 S259A S368A S368B S368D T143C T144A D500 D62 D683 D698 D699 R58 T792A T792B	
Renal disease		I12 I13 N00 N01 N02 N03 N04 N05 N07 N11 N14 N18 N19 Q61 N17	
Alcohol related disease		E244 E529a F105 F106 F107 F108 F109 G312 G621 G721 I426 K292 K70 K852 K860 l278a O354 P043	
Heart failure		I110 I130 I132 I420 I50 I426 I427 I429	
Diabetes	24900 24909 25008 25009	E10 E11	A10
Vascular disease		I700 I702 I708 I709 I739	
Hypertension*		I10 I11 I12 I13 I15	
COPD		J44	
Cancer		C	
Ischemic heart disease		I20 I21 I23 I24 I25	
Myocardial infarction		I21 I23	
VTE		H348e H348f I26 I636 I676 I801I802 I803 I808 I809 I81 I822 I823 I828 I829 O223 O225 O229 O871 O879 O873 O882 T817c T817d	
**Comedications, %(N)**			

© 2022 Søgaard M et al. *JAMA Network Open.*

Digoxin			C01AA05
Clopidogrel			B01AC04
Aspirin			B01AC06
Renin-angiotensin system inhibitors (ACEi/ARBs)			C09
Statins			C10
NSAID			M01AA M01AB M01AC M01AE M01AG M01AH M01AX01
Beta-blocker			C07
Calcium channel blocker			C07F C08 C09BB C09DB
Loop diuretics			C03C C03EB
Non-loop diuretics			C02DA C02L C03A C03B C03D C03E C03X C07C C07D C09BA C09DA 09XA52
Proton-pump inhibitors			A02BC
Antibiotics			J01

**We identified subjects with hypertension from diagnosis codes in the National Patient Registry and/or combination treatment with at least two of the following classes of antihypertensive drugs obtained from the Danish National Laboratory Registry:

I. Alpha adrenergic blockers (C02A, C02B, C02C)
II. Non-loop diuretics (C02DA, C02L, C03A, C03B, C03D, C03E, C03X, C07C, C07D, C08G, C09BA, C09DA, C09XA52)
III. Vasodilators (C02DB, C02DD, C02DG, C04, C05)
IV. Beta blockers (C07)
V. Calcium channel blockers (C07F, C08, C09BB, C09DB)
VI. Renin-angiotensin system inhibitors (C09).

© 2022 Søgaard M et al. *JAMA Network Open.*

eTable 2. Risk score definitions.

**Risk score**	**Points if present**
CHA_2DS_2-VASc[*]	
Congestive heart failure or Left Ventricular Dysfunction	1
Hypertension	1
Age 65-74 years	1
Age ≥ 75 years	2
Diabetes mellitus	1
Stroke (ischemic stroke, transient ischemic disease or systemic embolism)	2
Vascular disease (myocardial infarction, peripheral arterial disease, or aortic plaque)	1
Sex category (female)	1
HAS-BLED[†]	
Hypertension**	1
Abnormal renal function	1
Abnormal hepatic function	1
Stroke (ischemic stroke or transient ischemic attack)	1
Bleeding	1
Labile international normalized ratio[£]	1
Elderly age (> 65 years)	1
Drugs (aspirin, clopidogrel, or non-steroidal anti-inflammatory drugs)	1
Alcohol intake	1

[*]Reflects stroke risk in atrial fibrillation patients not in anticoagulant therapy (Lip GYH, Nieuwlaat R, Pisters R, Lane DA, Crijns HJGM. Refining clinical risk stratification for predicting stroke and thromboembolism in atrial fibrillation using a novel risk factor-based approach: the euro heart survey on atrial fibrillation. Chest 2010;137(2):263-72)

[†]Reflects bleeding risk in atrial fibrillation patients undergoing anticoagulant therapy (Pisters R, Lane DA, Nieuwlaat R, de Vos CB, Crijns HJGM, Lip GYH. A novel user-friendly score (HAS-BLED) to assess 1-year risk of major bleeding in patients with atrial fibrillation: the Euro Heart Survey. Chest 2010;138(5):1093-100).

[£]Not included due to unavailable information

****We identified subjects with hypertension from diagnosis codes in the National Patient Registry and/or combination treatment with at least two of the following classes of antihypertensive drugs obtained from the Danish National Laboratory Registry:

I. Alpha adrenergic blockers (C02A, C02B, C02C)

© 2022 Søgaard M et al. *JAMA Network Open.*

II. Non-loop diuretics (C02DA, C02L, C03A, C03B, C03D, C03E, C03X, C07C, C07D, C08G, C09BA, C09DA, C09XA52)
III. Vasodilators (C02DB, C02DD, C02DG, C04, C05)
IV. Beta blockers (C07)
V. Calcium channel blockers (C07F, C08, C09BB, C09DB)
VI. Renin-angiotensin system inhibitors (C09).

© 2022 Søgaard M et al. *JAMA Network Open.*

eTable 3. Results of sensitivity analyses among patients with new-onset atrial fibrillation with an indication for OAC therapy (i.e, intermediate or high baseline stroke risk).

Analyses/strata	Thromboembolism, %[#]		AF rehospitalization, %		OAC initiation, %		All-cause death, %	
	One-year follow-up	Three-year follow-up	One-year follow-up	Three-year follow-up	One-year follow-up	Three-year follow-up	One-year follow-up	Three-year follow-up
Stratification of baseline hazard function by inclusion period with interactions between inclusion period and all covariates								
Intermediate stroke risk	1.5	3.5	-*	-*	-*	-*	-*	-*
High stroke risk	2.8	5.1	-*	-*	-*	-*	-*	-*
Restriction of study period to 2014-2018								
Intermediate stroke risk	1.3	2.6[†]	26.0	23.2	20.8	31.1	29.6	48.7
High stroke risk	2.5	4.3[†]	35.8	33.3	17.0	21.6	33.0	60.0
Exclusion of patients with baseline aspirin treatment								
Intermediate stroke risk	1.6	3.5	-*	-*	-*	-*	-*	-*
High stroke risk	3.1	3.7	-*	-*	-*	-*	-*	-*

© 2022 Søgaard M et al. *JAMA Network Open.*

Death was treated as a competing risk. [#]Patients were censored if they started anticoagulation therapy and were weighted by the inverse of the probability of being censored. *IPCW methods were not used for assessment of secondary outcomes, because patients were not censored based on use of anticoagulants. [†]Follow-up for a maximum of three years or until administrative censoring at December 31, 2018.

© 2022 Søgaard M et al. *JAMA Network Open.*

eFigure. Flow diagram of the cohort creation process

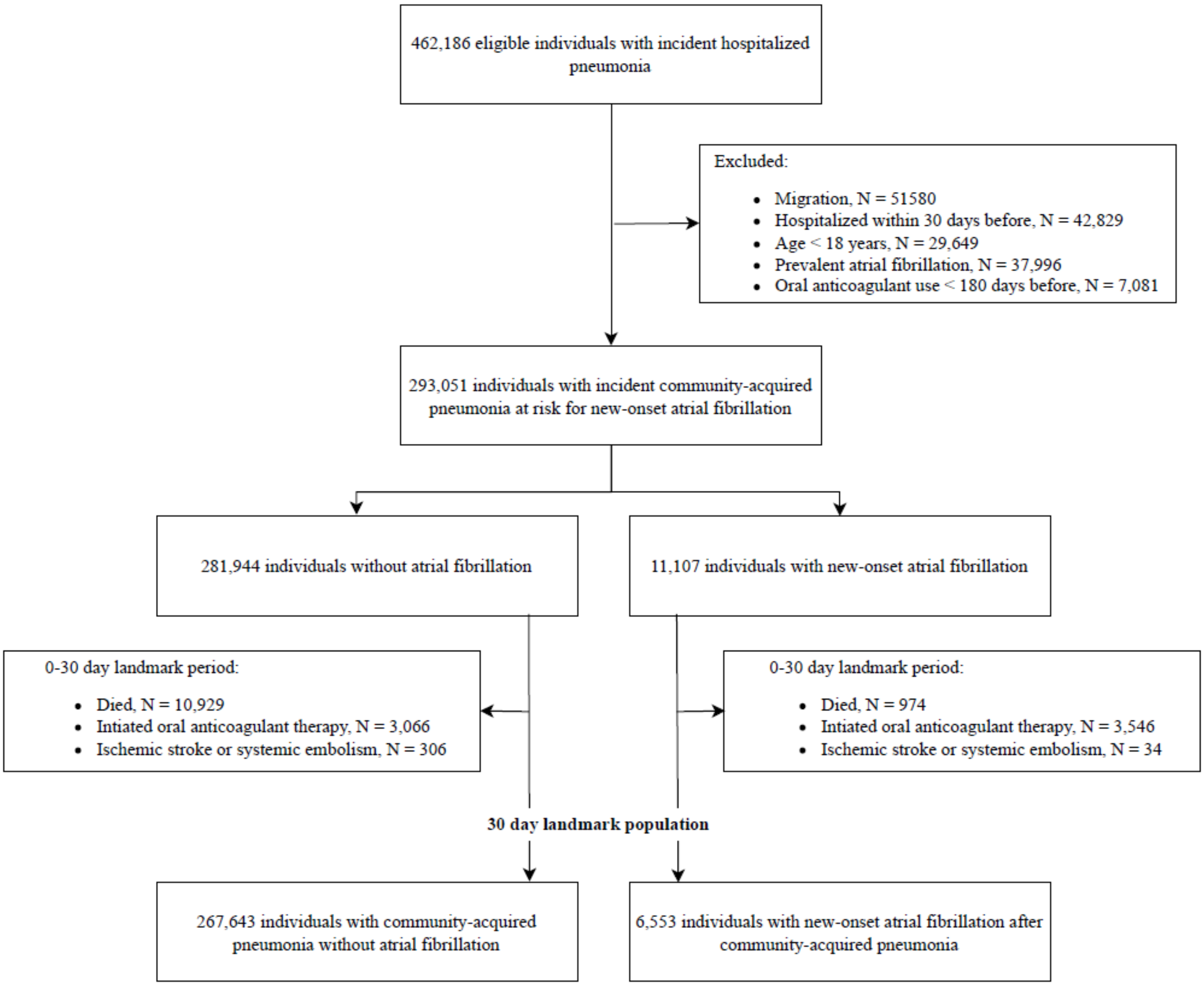

© 2022 Søgaard M et al. *JAMA Network Open.*
